# Multifactorial Exercise and Inflammatory Responses in Dementia: Findings from a Randomized Controlled Trial Secondary Analysis

**DOI:** 10.3390/life16081230

**Published:** 2026-07-25

**Authors:** Deborah A. Jehu, Mitchell Hanson, Ying Huang, Andre Soares, Colleen Hergott, Jennifer L. Waller, Lufei Young, William Hall, Dawnchelle Robinson-Johnson, Crystal Allen, Richard Sams, Ryan M. Carrick, Sadanand Fulzele, Mark Hamrick, Haidong Zhu, Yanbin Dong

**Affiliations:** 1 Community and Behavioral Health Sciences Department, Institute of Public and Preventive Health, School of Public Health, Augusta University, 2500 Walton Way, Augusta, GA 30904, USA; mihanson@augusta.edu (M.H.); ansoares@augusta.edu (A.S.); 2 Georgia Prevention Institute, Augusta University, 1499 Walton Way, Augusta, GA 30912, USA; yihuang@augusta.edu (Y.H.); hzhu@augusta.edu (H.Z.); ydong@augusta.edu (Y.D.); 3 Physical Therapy Department, College of Allied Health Sciences, Augusta University, 987 St. Sebastian Way, Augusta, GA 30912, USA; chergott@augusta.edu; 4 Department of Family and Community Medicine, Medical College of Georgia, Augusta University, 1120 15th St., HB 4000, Augusta, GA 30912, USA; 5 School of Nursing, University of North Carolina, 9201 University City Blvd., Charlotte, NC 28223, USA; lyoung57@charlottee.edu; 6 Claiborne Assisted Living Facility, Augusta University, 557 Furys Ferry Rd, Martinez, GA 30907, USA; whall@theclaiborne.com (W.H.); drobinsonjohnson@theclaiborne.com (D.R.-J.); 7 Georgia War Veterans Nursing Home, 1101 15th St., Augusta, GA 30901, USA; callen11@augusta.edu (C.A.); risams@augusta.edu (R.S.); 8 Department of Occupational Therapy, College of Allied Health Sciences, Augusta University, 987 St. Sebastian Way, Augusta, GA 30912, USA; rcarrick@augusta.edu; 9 Department of Neuroscience and Regenerative Medicine, Augusta University, 1462 Laney Walker Blvd., Augusta, GA 30912, USA; sfulzele@augusta.edu; 10 Department of Cellular Biology and Anatomy, Medical College of Georgia, Augusta University, 1120 15th St., CB 1101, Augusta, GA 30912, USA; mhamrick@augusta.edu

**Keywords:** inflammation, intervention, physical therapy, exercise, Alzheimer’s disease, dementia, cytokine, biomarker

## Abstract

The objective of this randomized controlled trial was to evaluate the impact of a multifactorial exercise program on circulating inflammation in people living with dementia (PWD) in residential care. This parallel-group, 6-month assessor-blinded trial (NCT05488951) allocated (1:1) 42 PWD to a multifactorial exercise intervention or usual care alone in residential care settings between July 2022 and January 2023. The exercise group engaged in 30 min of physical therapist-led strength and balance training, followed by 30 min of walking 3 x/week over 6 months, and received usual care. The usual care group only received care from healthcare providers, ongoing medical treatment, and opportunities to participate in social activities. Fasted blood was drawn at baseline and 6 months. Intention-to-treat (ITT) and per-protocol (PP; ≥2 x/week exercise vs. usual care) analyses were conducted. The ITT analysis revealed no differences between groups over time (*p* > 0.05). In adjusted PP analyses, there was a group-by-time interaction trend resulting in decreases in IL-1β (−3.6; +5.0 pg/mL) and IL-8 (−13.4; +14.0 pg/mL) in the exercise group compared with usual care (IL-1β: *p* = 0.089; IL-8: *p* = 0.087). Findings suggest adherence-dependent reductions in pro-inflammatory cytokines, indicating potential benefits of exercise. However, this trial was not powered for this secondary analysis, necessitating the need for larger, adequately powered trials.

## 1. Introduction

Dementia is a growing global public health priority, affecting approximately 55 million individuals worldwide, with nearly 10 million new cases annually [1]. By 2030, this number is projected to reach 75 million, imposing significant societal and economic burdens, such as global healthcare expenditures that exceeded USD 1.3 trillion in 2019 [1]. People living with dementia (PWD) face substantial health risks, including a two-to-three-fold increase in fall-related injuries, contributing significantly to healthcare costs, morbidity, and mortality [1,2,3]. The variable onset and diagnostic challenges of dementia progression have spurred research into additional diagnostic tools, such as blood biomarkers, to quantify functionality and improvement across therapeutic interventions aimed at enhancing quality of life in this population [4].

Inflammation contributes to the onset and progression of dementia [5,6,7,8,9,10,11]. Neuroinflammation, characterized by the release of pro-inflammatory markers like interleukin-1 (IL-1), IL-6, and tumor necrosis factor-alpha (TNFα) by activated glial cells, is implicated in multiple dementia types [12,13]. Elevated levels of several pro-inflammatory biomarkers have been detected in the cerebrospinal fluid and serum of individuals with mild to moderate cognitive decline [6,7]. Ultimately, higher levels of pro-inflammatory markers have been linked to poorer cognitive function, physical function, and increased fall frequency in older adults [8,9,10,11]. Because inflammatory processes typically involve dysregulation across multiple interconnected immune pathways rather than a single cytokine [14], evaluating a panel of circulating pro-inflammatory cytokines, anti-inflammatory cytokines, and chemokines may provide a more comprehensive assessment of the inflammatory response to exercise.

The World Health Organization’s global action plan for dementia emphasizes implementing lifestyle interventions, particularly exercise, to reduce cognitive impairment [15]. While there is no definitive disease-modifying therapy for all-cause dementia, exercise has shown promise in modulating key blood biomarkers linked to inflammation and neurodegeneration [16]. A lack of exercise has been linked to the chronic inflammation observed in dementia [17], while exercise has been shown to reduce systemic inflammation through alterations in anti-inflammatory cytokine expression, kynurenine metabolism, telomerase activity, and epigenetic mechanisms such as deoxyribonucleic acid methylation [18,19,20]. However, some research has shown greater inflammation following exercise in people with Alzheimer’s disease [21], which indicates that further work is needed in this area. Current research on exercise interventions in PWD predominantly features small sample sizes, with trials ranging from as few as 40 PWD [22] to larger groups of 68 PWD [23]. Most studies focus on unimodal aerobic exercise such as walking at moderate to high intensities, and lack diversity in exercise types [24,25]. Lastly, most exercise studies in PWD have focused on community-dwelling individuals or those receiving outpatient/day-care services, which may limit generalizability to individuals residing in long-term care settings who often experience greater cognitive and functional impairment [21,26,27]. This limitation underscores the critical need for broader, multifactorial exercise interventions to deepen our understanding of the physiological effects of exercise in PWD on inflammation. These efforts can drive improved outcomes and inform the design of therapeutic interventions for a population that has been historically excluded from research and underserved in healthcare access.

The aim of this secondary analysis of a randomized controlled trial (RCT) was to evaluate the effects of a multifactorial, physical therapist-led Otago Exercise Program versus usual care on a broad panel of inflammatory biomarkers in PWD residing in long-term care facilities. We hypothesized that the exercise intervention would modulate circulating inflammatory biomarkers to reflect improvements in systemic immune regulation compared with usual care.

## 2. Materials and Methods

### 2.1. Study Design

We used the strength and balance exercise from the executive function in people living with dementia (ENABLED) protocol in this pilot parallel, 6-month assessor-blinded RCT in a 1:1 ratio, with *n* = 21 participants having received exercise intervention plus usual care and *n* = 21 having received usual care alone. This randomized controlled trial was registered under clinicaltrials.gov (NCT05488951), and we published the protocol [28]. We used the Consolidated Standards of Reporting Trials (CONSORT) for quality of reporting.

### 2.2. Recruitment

The trial enrolled participants between July 2022 and January 2023. PWD were recruited from one local nursing home and one local assisted living facility. These local sites accommodated space for the blood draws and intervention.

### 2.3. Sample Size Estimation

The sample size of *n* = 42 PWD (21 participants/group) was determined using SAS 9.4 for the primary outcome (the Color-Word Stroop Test), as outlined in the ENABLED protocol [28]. While the trial was not specifically powered to detect statistically significant differences in inflammatory biomarkers, these outcomes were analyzed as secondary, exploratory endpoints.

### 2.4. Ethics Approval and Consent/Assent to Participate

Legally authorized representatives of eligible PWD were contacted by a study team member; they provided handwritten informed consent in person, via email, or by mail. Once informed consent was obtained, a study team member obtained handwritten assent from the PWD. All protocols have been approved by the Augusta University Institutional Review Board (Study #1836020).

### 2.5. Randomization and Blinding

In this pilot RCT, PWD were randomly assigned (1:1) to either the exercise group (*n* = 21) or the usual care group (*n* = 21). The statistician generated the randomization sequence using SAS 9.4, which was imported into OnCore. Randomization was stratified by biological sex (female/male) and study site, with block randomization performed using random block sizes of 2 and 4 within each group. Permutations of exercise or usual care were generated for each block.

### 2.6. Eligibility Criteria

The inclusion criteria included those who (1) were aged 55 years and older; (2) resided in a residential care facility; (3) had any type of dementia confirmed by medical records and/or a physician; (4) could read, write, and speak English with acceptable visual and auditory acuity; (5) were able to walk 4 m without the assistance of another person; (6) had a legally authorized representative who could provide informed consent; (7) were able to provide assent; (8) were able to understand and follow instructions; (9) had a life expectancy of ≥12 months as estimated by a healthcare provider; and (10) were able to stand unassisted for 30 s.

The exclusion criteria consisted of those (1) who were not able to comprehend or follow instructions, (2) with severe psychiatric conditions, (3) with delirium, (4) with an acute medical condition, (5) with a recent surgery impairing mobility, (6) who were enrolled in another research study, (7) with severe blindness, (8) with severe aphasia such that they were unable to perform assessments, (9) receiving hospice care, and (10) with a medical condition precluding exercise (e.g., unstable cardiac disease).

### 2.7. Demographics

To describe participants and control for confounding factors, we collected demographic variables such as age, sex, race, medication details, dementia type, and time since dementia onset from PWD’ medical charts. We used the Functional Comorbidity Index, including 18 evenly weighted comorbidities. The total number of comorbidities (0–18) was obtained from participants’ medical charts. We also measured fall risk utilizing the Morse Fall Scale, a widely utilized tool in clinical settings [29]. It evaluates several factors, including history of falling, secondary diagnoses, ambulatory aid use, intravenous therapy, gait, and mental status. Scores range from 0 to 125, with higher scores indicating greater fall risk. Fall history over the past six months was also extracted from incident reports documented in participants’ medical charts. Lastly, we utilized the Montreal Cognitive Assessment (MoCA) to measure global cognition in this RCT [30]. The MoCA also measures executive function, short-term memory recall, visuospatial abilities, attention, concentration and working memory, language, and orientation to time and place.

### 2.8. Plasma Cytokine Analysis

Fasted blood samples of 16 mL were collected from PWD by the study nurse at baseline and 6 months. The samples were processed within 2 h of collection to isolate plasma and serum, which were subsequently stored at −80 °C until analysis. Inflammatory blood biomarkers were analyzed with the LEGENDplex Human Inflammaging Panel1 13-Plex (BioLegend, San Diego, CA, USA), which included IL-1β, IL-6, IL-8, IL-10, IL-12p70, IL-17A, IL-18, IL-23, IL-33, IFN-α2, IFN-γ, TNF-α, and MCP-1. This panel was selected to capture multiple inflammatory pathways implicated in dementia [13,31], including innate and adaptive immune signalling, chemotaxis, and anti-inflammatory regulation, rather than focusing on individual cytokines. Plasma samples were thawed, mixed, centrifuged to remove particulates, and diluted 2-fold with assay buffer. Samples and standards (prepared with matrix solution) were incubated overnight at 4 °C with capture beads, washed, and treated with detection antibodies, followed by SA-PE incubation. Data acquisition was performed on a Novocyte Quanteon flow cytometer (Agilent Technologies, Santa Clara, CA, USA), with standard curves and protein concentrations calculated using the R package DrLumi (version 0.1.2; ISGlobal, Barcelona, Spain).

### 2.9. Exercise (Intervention)

The original OEP is a strength and balance training program led by a physical therapist, designed for frail older adults living in the community and supported by evidence-based practices [32,33]. We adapted the OEP among 21 PWD per the ENABLED protocol [28]. For our trial, a physical therapist led 30 min of individually tailored lower-body strength and balance exercises from the OEP manual, followed by 30 min of walking 3 x/week for 6 months in groups of 5–7 PWD randomized to the exercise group. The physical therapist individually progressed the exercise program throughout the 6-month intervention based on each participant’s functional abilities and health status. When participants experienced declines in health or function, the physical therapist modified the program to meet their current needs, and as participants improved and became stronger, the program was adjusted to provide appropriate progression and continued challenge. The only equipment needed for this feasible program was ankle weights and chairs. The physical therapist and/or study team members walked with PWD to the exercise class from their room and escorted them back after class to foster adherence. Exercise classes were offered around meal and bathing times, in coordination with the residential facilities and other regular activities. Adherence was tracked by the physical therapist at each exercise session.

### 2.10. Usual Care (Control)

All 42 PWD were provided with usual care from their health care providers, including specialist and local doctor visits, community nurse visits, paid care provider visits, hospitalizations as required, and any ongoing treatment for any illness and/or comorbidities. The research team did not interfere with ongoing medical treatment (including changes in medication or prescribed physical therapy) in either the exercise or usual care group, and such changes were not documented as part of the study. No structured exercise program, mobility training, or rehabilitation program was otherwise provided at either residential care facility, other than any ongoing prescribed treatment. Both groups were provided with the opportunity to engage in ongoing social activities at the nursing home or assisted living facility (e.g., bingo, outings, arts and crafts).

### 2.11. Statistical Analysis

All statistical analyses were conducted using SAS 9.4, with statistical significance assessed at an alpha level of 0.05 unless otherwise specified. Descriptive statistics were calculated within each group (exercise and usual care) and measurement time (baseline and six months), where appropriate. Assumptions for inferential statistical methods were evaluated before testing, including expected values greater than 5 for chi-square tests, and normality of residuals and equality of variance for analysis of variance. Missing data were not imputed; instead, mixed model methods were applied.

Two sets of analyses were performed: an intention-to-treat (ITT) analysis, which included all PWD, and a per-protocol (PP) analysis, which included all usual-care PWD and only those in the exercise group who attended at least two sessions per week for six months. For variables measured only at baseline or at six months, chi-square tests or two-sample *t*-tests were used to evaluate group differences. To assess changes over time between groups, mixed models unadjusted for covariates were utilized. Fixed effects included group, time, and their interaction, while PWD nested within group was treated as a random effect. A compound symmetric correlation structure was used to estimate correlations between time points. F-tests for all effects were performed with a Kenward–Roger adjustment to the denominator degrees of freedom. The primary statistical test was the F-test for the group-by-time interaction, with significance indicating differing changes over time between the exercise and usual-care groups. Bonferroni adjustments were applied to control the overall alpha level for pairwise comparisons of interest. Subsequent analyses adjusted for covariates such as age, sex, and MoCA scores, with the latter treated as a time-dependent covariate. Adjusted analyses were considered the primary analyses because they accounted for prespecified covariates expected to influence inflammatory outcomes and provided more precise estimates of intervention effects. Unadjusted analyses were conducted as complementary analyses to evaluate the robustness of the findings.

## 3. Results

### 3.1. Participant Characteristics

**Figure 1:**
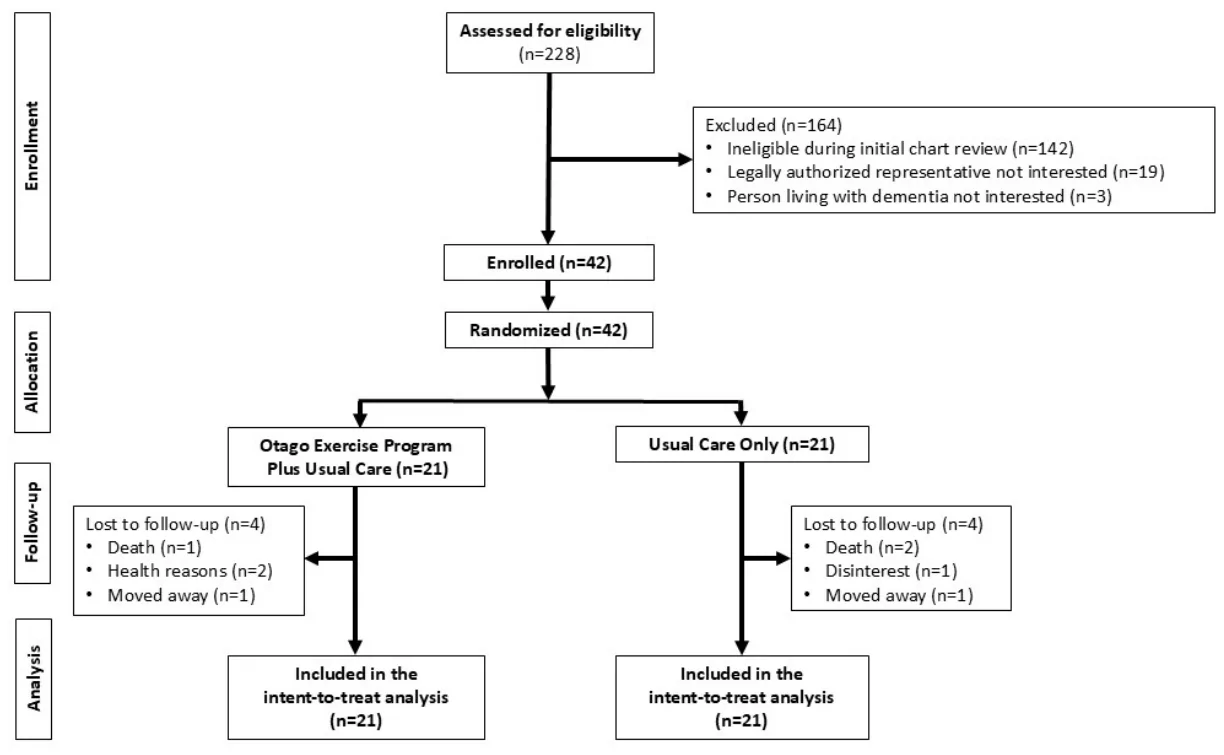
Study flow diagram using the ENABLED trial protocol [34].

Participant characteristics for the ITT and PP are summarized in Table 1. The participant flow diagram is shown in Figure 1 and has been previously reported [34]. The mean age of PWD was 82.1 ± 8.1 years, with 35.7% identifying as female, and 83% white. The average time since dementia onset was 3.6 ± 5.3 years. Most PWD had unspecified dementia (50%), and Alzheimer’s disease was the next most common diagnosis (38.1%). PWD resided in various living settings: 57.1% in nursing homes, 16.7% in memory care facilities, and 26.2% in assisted living. The mean Morse Fall Scale score was 45.6 ± 23.9 points, and the mean MoCA score was 10.0 ± 5.9 points. The systolic and diastolic blood pressure of the exercise group was 146 ± 29.8/79.8 ± 12.5 mmHg at baseline and 144 ± 17.8/83.2 ± 12.8 mmHg at follow-up, and the usual-care group had 149 ± 18.7/81.9 ± 13.9 mmHg at baseline and 144 ± 18.0/80.5 ± 9.6 mmHg at follow-up. Exercise adherence was moderate, with participants completing an average of 60.2% ± 34.5% of the prescribed exercise sessions, corresponding to 47 of the 78 scheduled training hours.

### 3.2. Inflammatory Blood Biomarkers

**Figure 2:**
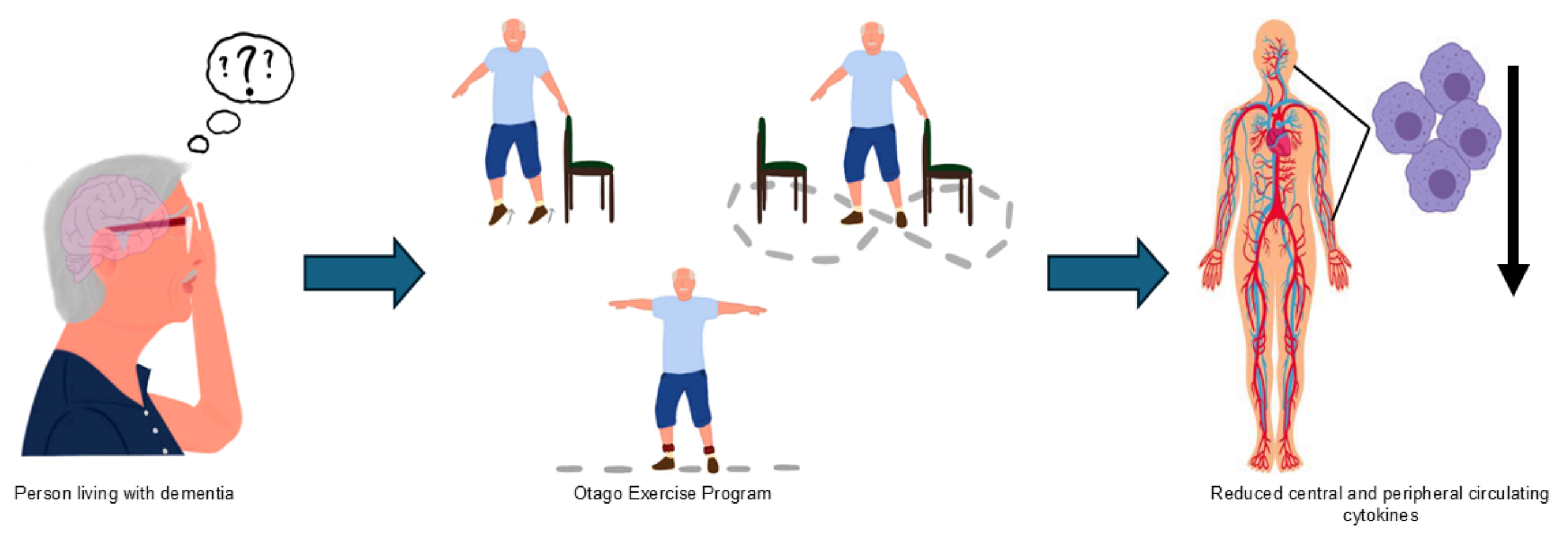
Conceptual schematic: The Otago Exercise Program may modify central and peripheral inflammation in PWD in residential care facilities.

Both unadjusted (Supplementary Table S1) and adjusted ITT and PP analyses (Table 2) showed no significant differences between the OEP and usual-care groups. In the adjusted PP analysis, the exercise group demonstrated a trend towards reduced levels, and the usual-care group exhibited increased levels of IL-1β (*F* = 3.28, *p* = 0.089) and IL-8 (*F* = 3.37, *p* = 0.087) from baseline to 6 months. A conceptual model is depicted in Figure 2.

## 4. Discussion

This RCT evaluated the effects of a multifactorial exercise intervention comprising strength training, balance exercises, and walking on several inflammatory biomarkers associated with dementia. In contrast to previous trials that have examined unimodal exercise interventions in PWD or have primarily enrolled community-dwelling or outpatient individuals with mild cognitive impairment [24,25,35,36], this trial is the first to implement a multifactorial exercise program in a diverse cohort of PWD residing in residential care facilities—a population likely to experience more severe cognitive and physical impairments. Although no significant between-group differences in inflammatory biomarkers were observed in the primary analyses, the PP analyses revealed group-by-time trends, with two pro-inflammatory cytokines (IL-1β and IL-8) decreasing in the exercise group and increasing in the usual care group. These trends warrant further investigation into the potential effects of multifactorial exercise on inflammatory processes in a heterogeneous sample of PWD residing in residential care facilities. The absence of changes across the full biomarker panel may reflect the complexity and heterogeneity of inflammatory regulation in PWDs, where individual immune pathways may respond differently depending on disease type and stage, baseline inflammation, exercise exposure, and other clinical factors [13,31,37], warranting larger future trials.

Chronic inflammation is increasingly recognized as a key contributor to neurodegenerative conditions, including dementia [17]. The exploratory trends observed in the present trial are consistent with the limited body of evidence suggesting that exercise may favorably influence inflammatory markers in PWD [35,38]. Several possible mechanisms have been proposed to explain these trends. Exercise may reduce inflammation by decreasing pro-inflammatory cytokines (e.g., IL-1β, IL-6, and TNF-α), suppressing microglial activation through pathways such as nuclear factor-kappa B and Jun N-terminal kinase, increasing anti-inflammatory neurotrophic factors such as BDNF, and promoting the release of anti-inflammatory mediators, including IL-10 and IL-1RA [39]. Evidence from both animal and human studies further suggests that exercise may attenuate inflammation within the central nervous system as well as in the periphery, with peripheral inflammatory processes potentially influencing brain function through circulating cytokines that access the central nervous system via circumventricular organs or a compromised blood–brain barrier [39]. Despite these plausible biological mechanisms, the present trial observed only trends toward reductions in inflammatory markers. One possible explanation is that our intervention was multifactorial, incorporating strength, balance, and walking exercises, whereas previous studies reporting significant reductions in inflammation have primarily focused on aerobic exercise [35,38]. Given the limited guidance regarding optimal exercise prescription for PWD [40], factors such as exercise type, dose, intensity, and duration may require further refinement to elicit measurable biological changes. Notably, the trends toward reduced pro-inflammatory cytokines were observed only in the adjusted PP analyses, suggesting that the type of exercise, as well as greater adherence and cumulative exercise exposure, may influence inflammatory outcomes in PWD. Participants who consistently attended exercise sessions likely received a greater overall dose of the intervention, which may be necessary to achieve measurable changes in inflammatory markers, whereas lower adherence in the ITT analysis may have diluted the potential biological effects of exercise. Nevertheless, these trends should be viewed as exploratory, as only *n* = 9/21 PWD adhered to ≥2/3 sessions/week over 6 months. Large-scale trials are warranted to validate the findings.

Our findings should also be considered within the broader context of the ENABLED trial. The adapted OEP was shown to be feasible and acceptable [34] and resulted in improvements in working memory, leg strength, and quality of life compared with usual care [34,41]. However, no significant differences were observed in metabolic, cellular, or epigenetic biomarkers [42]. Together, these findings suggest that functional benefits may occur in the absence of detectable changes in biological markers and highlight the need for further research to clarify the potential relationship between exercise and inflammatory processes in PWD.

### 4.1. Limitations

Some limitations of this study should be considered. Because this pilot was powered for executive function rather than biomarker outcomes [28], it may have been underpowered to detect small-to-moderate biological changes in secondary biomarker endpoints, particularly in the PP analysis with *n* = 9/21 adherent PWD. The participant profile reflects real-world diversity in dementia care; however, it also brings considerable heterogeneity. All participants received usual care from their healthcare providers, but the type of usual care was not documented (e.g., some may have received physical therapy). Additionally, differences in inflammation between groups may, in part, be due to differences in attention bias, cognitive and physical frailty, therapist contact, increased motivation, or enhanced social engagement. Although we applied a Bonferroni correction for multiple comparisons, a false-positive finding remains possible.

### 4.2. Implications

Trends in reduced pro-inflammatory cytokines among adherent PWD may suggest that sustained and tailored exercise programs could offer therapeutic benefits in reducing inflammation in PWD. It is possible that optimizing adherence to exercise (i.e., attending ≥ 2 exercise classes per week) may elicit stronger anti-inflammatory and neuroprotective effects than low adherence; however, this should be confirmed in a larger sample. This trial also highlights the inherent challenges in conducting research with PWD, such as adherence. Few trials have included PWD due to researchers’ eligibility criteria [28,43]. Our findings highlight the need for larger, more diverse trials with improved statistical power, enhanced participant adherence, and higher-intensity, longer-duration exercise interventions to better detect biomarker changes and inform targeted, evidence-based strategies to improve cognitive health and quality of life, and reduce health disparities among PWD. Efforts to explore how exercise impacts dementia-related biomarkers can guide targeted therapies, clinical protocols, and lifestyle interventions.

## 5. Conclusions

While group differences were not statistically significant, this secondary analysis of an RCT provides preliminary evidence that multifactorial exercise trended to decrease inflammatory cytokines in a heterogeneous cohort of PWD, but only in the adjusted PP analyses among those who adhered to exercise at least 2 x/week. Therefore, these findings are exploratory and highlight the need for larger, adequately powered trials focused on inflammatory outcomes.

## Figures and Tables

**Table 1:** Baseline descriptive statistics of PWD in the exercise and usual-care group; mean ± standard deviation (range) [34].

Descriptive Measure	All Participants (*n* = 42)	Exercise (ITT: *n* = 21)	Exercise (PP: *n* = 9)	Usual Care (*n* = 21)
Age, years	82.1 ± 8.10 (60–98)	80.7 ± 7.50 (68.0–94.0)	81.9 ± 8.3 (68.0–93.0)	83.5 ± 8.6 (60.0–98.0)
Female sex, *n* (%)	15.0 (35.7)	7 (33.3)	4 (44.4)	8 (38.1)
Race: Black, White, *n* (%)	7.0 (16.7), 35 (83.3)	4 (9.50), 17 (40.5)	2 (22.2), 7 (77.8), 0 (0)	3 (7.10), 18 (42.9)
BMI, kg/m^2^	26.6 ± 5.50 (17.6–41.8)	27.0 ± 5.7 (17.6–36.2)	28.0 ± 6.5 (19.2–36.2)	26.2 ± 5.5 (19.1–41.8)
Dementia type, *n* (%)				
Alzheimer’s disease	16 (38.1)	7 (33.3)	3 (33.3)	9 (42.9)
Vascular dementia	3 (7.10)	1 (4.80)	0 (0.0)	2 (9.50)
Parkinson’s disease Dementia	1 (2.40)	1 (4.80)	1 (11.1)	0 (0.0)
Alcohol-induced dementia	1 (2.4)	0 (0.0)	0 (0.0)	1 (4.80)
Unspecified, without Behavioral disturbance	21 (50.0)	12 (57.1)	5 (55.6)	9 (42.9)
Dementia onset, years	3.50 ± 5.2 (0.2 to 26.6)	2.1 ± 1.5 (0.4 to 5.2)	1.4 ± 1.2 (0.4 to 3.0)	5.0 ± 7.2 (0.2 to 26.6)
FCI, total number	3.70 ± 2.0 (1–9)	4.0 ± 2.1 (1–8)	3.6 ± 2.5 (1–8)	3.4 ± 2.0 (1–9)
Medications, total number	12.5 ± 5.6 (0–26)	13.6 ± 6.3 (6–26)	14.7 ± 6.7 (6–26)	11.4 ± 4.8 (0–24)
Fall history in the last 6 months	2.8 ± 12.2 (0–78)	4.3 ± 16.9 (0–78)	0.4 ± 0.7 (0–2)	1.3 ± 2.3 (0–9)
Morse Fall Scale, points	45.6 ± 23.9 (15–90)	47.6 ± 24.9 (15–90)	46.1 ± 22.6 (15–80)	43.6 ± 23.4 (15–80)
Site				
Nursing home, *n* (%)	24 (57.1)	13 (31.0)	5 (55.6)	11 (26.2)
Memory care, *n* (%)	7 (16.7)	5 (11.9)	3 (33.3)	2 (4.80)
Assisted living, *n* (%)	11 (26.2)	3 (7.10)	1 (11.1)	8 (19.0)
MoCA, points	10.0 ± 5.9 (0–19)	10.6 ± 6.3 (0–19)	9.3 ± 7.4 (0–18)	9.5 ± 5.5 (1–19)

BMI—body mass index; ; FCI—Functional Comorbidity Index; MoCA—Montreal Cognitive Assessment; PWD—people with dementia.

**Table 2:** Adjusted changes in circulating interleukins, cytokines, and chemokines over time in PWD in residential care facilities for the ITT and PP analyses, controlling for age, sex, and the MoCA score; mean (SE).

Inflammatory Biomarker	ITT Least Squares Mean (SE) for Group × Time Interaction					
	Exercise (*n* = 21)		Usual Care (*n* = 21)		*F*	*p*-Value
	Baseline	6 Months	Baseline	6 Months		
IL-1β (pg/mL)	6.70 (2.50)	5.90 (2.50)	11.8 (4.80)	16.5 (5.30)	1.29	0.277
IL-6 (pg/mL)	13.3 (4.90)	21.4 (5.50)	16.4 (6.40)	28.0 (6.80)	0.17	0.685
IL-8 (pg/mL)	38.0 (16.3)	30.3 (17.1)	89.0 (33.6)	101 (34.1)	2.01	0.181
IL-10 (pg/mL)	10.3 (4.20)	12.0 (4.30)	12.1 (3.20)	13.1 (3.30)	0.02	0.883
IL-12p70 (pg/mL)	8.20 (0.80)	7.00 (0.90)	9.20 (3.00)	9.60 (3.10)	1.00	0.332
IL-17A (pg/mL)	2.60 (0.90)	2.60 (0.90)	3.50 (1.50)	3.80 (1.50)	0.21	0.658
IL-18 (pg/mL)	556 (118)	539 (137)	768 (136)	699 (142)	0.05	0.823
IL-23 (pg/mL)	24.3 (10.2)	20.0 (10.3)	27.2 (12.0)	27.4 (12.2)	0.96	0.340
IL-33 (pg/mL)	243 (95.7)	197 (97.6)	362 (87.0)	346 (91.2)	0.15	0.700
IFN-α2 (pg/mL)	12.0 (4.50)	11.4 (4.50)	13.9 (5.30)	14.1 (5.40)	0.31	0.586
IFN-γ (pg/mL)	9.30 (3.90)	12.1 (4.20)	14.3 (5.10)	18.9 (5.80)	0.08	0.778
TNF-α (pg/mL)	37.9 (12.7)	30.1 (12.9)	48.2 (150)	45.1 (15.3)	0.27	0.612
MCP-1 (pg/mL)	126 (19.2)	108 (19.6)	99.9 (15.3)	98.7 (16.1)	0.68	0.416
	**PP Least Squares Mean (SE) for Group × Time Interaction**					
	**Exercise (** ***n*** ** = 9)**		**Usual Care (** ***n*** ** = 21)**			
IL-1β (pg/mL)	9.73 (5.40)	6.16 (5.34)	10.5 (5.55)	15.5 (5.86)	3.28	**0.089**
IL-6 (pg/mL)	13.5 (4.89)	16.8 (5.28)	16.1 (6.54)	27.8 (6.95)	1.08	0.311
IL-8 (pg/mL)	39.3 (13.9)	25.9 (16.4)	79.0 (30.3)	93.0 (30.6)	3.37	**0.087**
IL-10 (pg/mL)	9.34 (6.86)	16.4 (6.88)	11.8 (3.31)	12.5 (3.38)	0.75	0.410
IL-12p70 (pg/mL)	NE	NE	NE	NE	NE	NE
IL-17A (pg/mL)	3.42 (1.36)	3.28 (1.36)	4.02 (1.62)	4.33 (1.65)	0.40	0.544
IL-18 (pg/mL)	574 (153)	573 (162)	789 (151)	722 (157)	0.06	0.800
IL-23 (pg/mL)	32.2 (15.7)	27.0 (15.7)	30.6 (14.0)	30.6 (14.1)	0.93	0.348
IL-33 (pg/mL)	247 (117)	322 (151)	367 (84.0)	367 (87.0)	0.19	0.668
IFN-α2 (pg/mL)	10.8 (5.05)	9.76 (5.11)	13.2 (5.39)	13.7 (5.40)	0.75	0.402
IFN-γ (pg/mL)	11.0 (5.93)	18.5 (6.67)	14.7 (5.88)	18.7 (6.44)	0.21	0.656
TNF-α (pg/mL)	43.6 (21.1)	34.4 (21.4)	51.8 (17.7)	49.7 (18.0)	0.42	0.529
MCP-1 (pg/mL)	117 (13.3)	97.7 (14.1)	96.7 (14.8)	95.0 (15.7)	0.70	0.411

IFN—interferon; ITT—intention to treat; PP—per protocol; IL—interleukin; MCP-1: monocyte chemoattractant protein-1; MoCA: Montreal Cognitive Assessment; NE: not estimable due to small sample size; SE—standard error; TNF: tumor necrotic factor.

## Data Availability

The original contributions presented in this study are included in the article/Supplementary Material. Further inquiries can be directed to the corresponding author.

## Supplementary Materials

The following supporting information can be downloaded at: https://www.mdpi.com/article/10.3390/life16081230/s1, Table S1: Unadjusted changes in circulating interleukins, cytokines, and chemokines over time in people living with dementia in residential care facilities; mean (SE).

## Abbreviations

The following abbreviations are used in this manuscript:

PWD	People living with dementia
ITT	Intention to treat
PP	Per protocol
RCT	Randomized controlled trial
OEP	Otago Exercise Program
IL	Interleukin
TNF	Tumor necrosis factor
BDNF	Brain-derived neurotrophic factor
IFN	Interferon
MCP	Monocyte chemoattractant protein
