# Supplementary material for: Multifactorial Exercise and Inflammatory Responses in Dementia: Findings from a Randomized Controlled Trial Secondary Analysis
Source: Life (Basel). 2026 Jul 25;16(8):1230. doi: 10.3390/life16081230 (PMC13514734; doi:10.3390/life16081230)
Supplement: Supplementary file 1 [file life-16-01230-s001.zip › life-4412433-supplementary.pdf]

Table S1. Unadjusted changes in circulating interleukins, cytokines, and chemokines over time in people living with dementia in residential care facilities; mean (SE)

Inflammatory Biomarker	ITT Least Squares Mean (SE) for Group x Time Interaction					
	Exercise (n=21)		Usual Care (n=21)		F	p-value
	Baseline	6 months	Baseline	6 months		
IL-1β (pg/ml)	5.80 (1.80)	5.60 (1.90)	12.3 (4.20)	16.3 (4.70)	0.84	0.376
IL-6 (pg/ml)	14.7 (3.40)	22.9 (3.80)	19.0 (5.60)	32.2 (6.00)	0.35	0.556
IL-8 (pg/ml)	52.1 (11.8)	46.5 (12.1)	106 (30.5)	116.2 (31.1)	1.36	0.263
IL-10 (pg/ml)	10.5 (3.40)	12.5 (3.60)	12.0 (2.50)	13.3 (2.70)	0.02	0.884
IL-12p70 (pg/ml)	7.50 (1.30)	6.60 (1.40)	8.50 (2.70)	9.1 (2.80)	0.81	0.382
IL-17A (pg/ml)I(	2.70 (0.60)	2.70 (0.60)	3.8 (1.40)	4.0 (1.40)	0.15	0.702
IL-18 (pg/ml)	485 (36.8)	438 (37.9)	716 (117)	640 (127)	0.03	0.868
IL-23 (pg/ml)	26.1 (6.60)	22.4 (6.70)	30.3 (9.7)	30.4 (9.80)	0.82	0.376
IL-33 (pg/ml)	324 (71.1)	274 (75.0)	413 (55.7)	389 (60.7)	0.12	0.727
IFN-α2 (pg/ml)	11.9 (3.50)	11.0 (3.50)	12.8 (4.00)	12.8 (4.00)	0.28	0.604
IFN-γ (pg/ml)	9.70 (2.60)	12.0 (2.80)	15.1 (4.50)	19.9 (5.40)	0.17	0.690
TNF-α (pg/ml)	34.6 (8.90)	30.1 (9.20)	46.7 (11.0)	42.6 (11.4)	0.00	0.961
MCP-1 (pg/ml)	138 (16.0)	115 (16.5)	112 (12.7)	106.4 (13.8)	0.87	0.360
	**Per Protocol Least Squares Mean (SE) for Group x Time Interaction**					
	**Exercise (n=9)**		**Usual Care (n=21)**			
IL-1β (pg/ml)	7.21 (4.12)	6.74 (4.14)	12.3 (4.25)	16.31 (4.72)	0.92	0.353
IL-6 (pg/ml)	15.42 (3.02)	18.8 (3.02)	19.0 (5.59)	32.18 (6.02)	1.59	0.220
IL-8 (pg/ml)	44.4 (13.7)	44.97 (14.0)	106 (26.9)	102.75 (30.3)	0.02	0.899
IL-10 (pg/ml)	10.4 (6.30)	16.7 (6.30)	12.0 (2.48)	13.33 (2.68)	0.38	0.555
IL-12p70 (pg/ml)	8.63 (2.30)	7.52 (2.31)	8.55 (2.65)	9.09 (2.77)	0.91	0.352
IL-17A (pg/ml)	2.75 (0.85)	2.62 (0.85)	3.79 (1.38)	4.03 (1.40)	0.35	0.565
IL-18 (pg/ml)	484 (64.6)	452 (64.6)	715 (137)	640.17 (148)	0.05	0.827
IL-23 (pg/ml)	30.0 (12.7)	25.4 (12.7)	30.3 (9.72)	30.37 (9.82)	0.91	0.352
IL-33 (pg/ml)	300 (99.2)	398 (121)	413 (53.6)	399.52 (57.7)	0.48	0.499
IFN-α2 (pg/ml)	11.5 (4.19)	10.7 (4.21)	12.8 (3.97)	12.76 (4.01)	0.21	0.653
IFN-γ (pg/ml)	10.4 (4.68)	16.1 (5.10)	15.1 (4.49)	19.87 (5.43)	0.01	0.909
TNF-α (pg/ml)	36.1 (16.2)	33.6 (16.4)	46.8 (11.0)	42.64 (11.4)	0.03	0.870
MCP-1 (pg/ml)	126 (12.5)	103 (12.5)	112 (12.7)	106.37 (13.8)	0.84	0.369

IFN-Interferon, IL- Interleukin; MCP-1: Monocyte Chemoattractant Protein-1; SE-Standard Error; TNF: Tumor Necrotic Factor
